# Ideal treatment strategy for chylous mesenteric cyst: a case report

**DOI:** 10.1186/s13256-018-1716-x

**Published:** 2018-10-17

**Authors:** Daniel Paramythiotis, Petros Bangeas, Anestis Karakatsanis, Alexandros Iliadis, Georgia Karayannopoulou, Antonios Michalopoulos

**Affiliations:** 1 1st Propedeutic Surgical Unit, AHEPA University Hospital of Thessaloniki, St Kiriakidi 1, 54621 Thessaloniki, Greece; 2 Pathology Department, AHEPA University Hospital of Thessaloniki, Thessaloniki, Greece

**Keywords:** Chylous mesenteric cyst, Lymphatic cyst, Ultrasound, Computed tomography, Laparotomy

## Abstract

**Background:**

A mesenteric chylous cyst is defined as a cyst occurring in the mesentery of the gastrointestinal tract anywhere from the duodenum to the rectum and is diagnosed most often during the fifth decade of life.

**Case presentation:**

In our case report, we describe a case of 38-year-old Greek woman who presented at our Emergency Department complaining of abdominal pain without any other symptoms. Her medical and family histories were clear and she had never had any abdominal interventions. During an imaging examination with ultrasound of her abdomen, an anechoic lesion in her upper left abdomen was revealed. In a further investigation with computed tomography, a well-defined hypodense cystic 7.08 × 6.05 cm mass with mild enhancement was noted. The mass was excised by open laparotomy within healthy borders and the specimen was sent for pathological examination. The histopathological findings were found to be most consistent with a simple lymphatic (chylous) cyst of the mesentery. A review of the literature considering this rare entity was also performed to evaluate our treatment strategy and the result was analyzed.

**Conclusions:**

Chylous cysts represent a diagnostic challenge and they should be considered when a physician encounters an intraabdominal mass. Physical examination and imaging do not always provide a diagnosis and surgical management should be advised due to the potential complications that may develop.

## Background

**Fig. 1:**
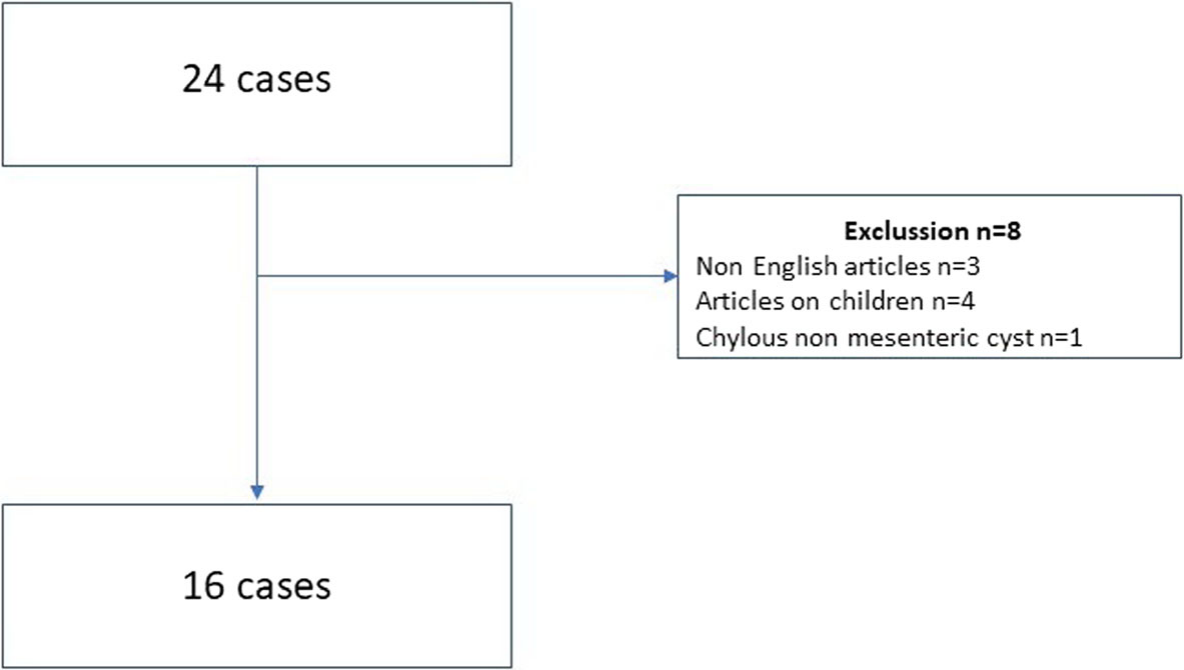
PRISMA Chart with the exclusion articles

Mesenteric cysts represent a rare pathologic entity. Their existence was first reported in 1507 by Benevieni, an Italian anatomist, when he performed an autopsy on an 8-year-old boy and the first effective surgical excision was performed in 1880 by Tillaux [1, 2]. Mesenteric cysts are identified in approximately 1 out of 100,000 adult hospital admissions [1] and they are most frequently located in the small bowel mesentery (ileum in 60%), followed by the large bowel mesentery (ascending colon in 24%), the retroperitoneum (14.5%), and the omentum [3]. A mesenteric chylous cyst is defined as a cyst in the mesentery of the gastrointestinal tract anywhere from the duodenum to the rectum, and which may extend from the base of the mesentery into the retroperitoneum [4]. In addition, a chylous or lymphatic cyst is a rare subclassification of mesenteric cysts, specifically of lymphatic origin and usually represents as benign lesions [5]. Chylous cysts represent approximately 7.3% of all abdominal cysts and they were first described by Rokitansky in 1842 [6]. These cysts are diagnosed most often during the fifth decade of life and they affect both sexes equally [4]. The pathogenesis remains unclear and there are several theories that have been proposed for the formation of these cysts. One of them suggested that they represent benign proliferations of ectopic lymphatics, which lack communication with the main lymphatic system. Another theory proposed that embryonic lymphatic channels gradually become enlarged, due to the failure of joining the venous system. Chylous cysts may also result from trauma to the lymphatic channels. Finally, it has been proposed that non-fusion of the leaves of mesentery results in accumulation of lymphatic fluid within this space [5]. Mesenteric cysts usually reveal nonspecific clinical and imaging findings [1–5]. The treatment of choice is complete surgical removal [5–7]. In this study, we analyze the treatment strategy of chylous mesenteric cyst in a woman. A review of the literature considering this rare entity is also performed to evaluate our treatment strategy. A literature search through MEDLINE database was performed. The search terms employed were “Chylous mesenteric cyst” and “Lymphatic mesenteric cyst.” The search covered the period from January 2000 until June 2018, and from an initial 24 cases, eight were rejected (Fig. 1). Descriptive statistics were used appropriately. Statistical analysis was performed in SPSS version 23 (SPSS Inc, Chicago, IL, USA).

## Case presentation

**Fig. 2:**
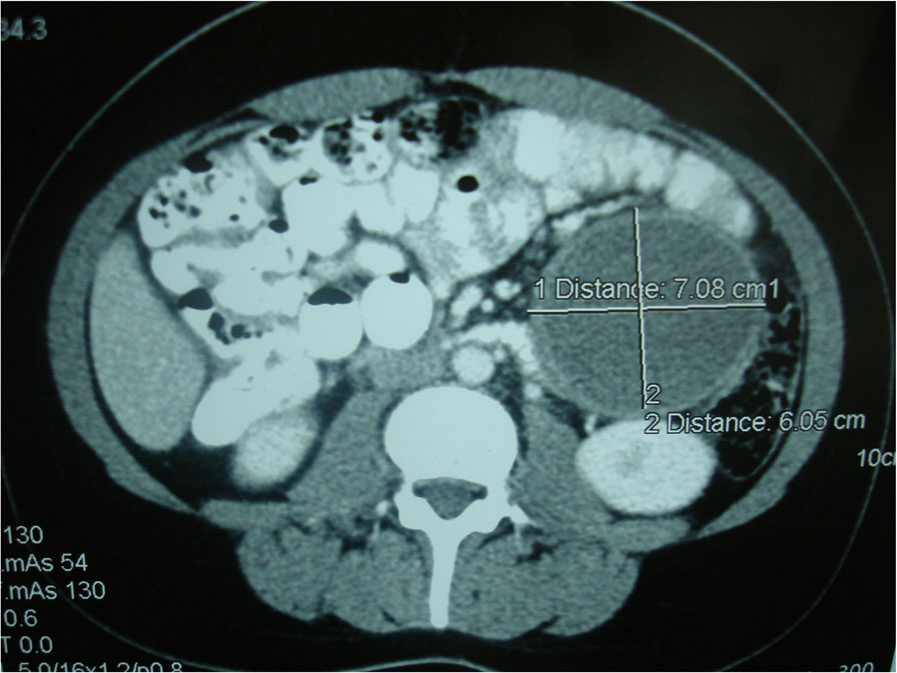
Computed tomography showed a well-defined hypodense cystic 7.08 × 6.05 cm chylous cyst with mild enhancement

**Fig. 3:**
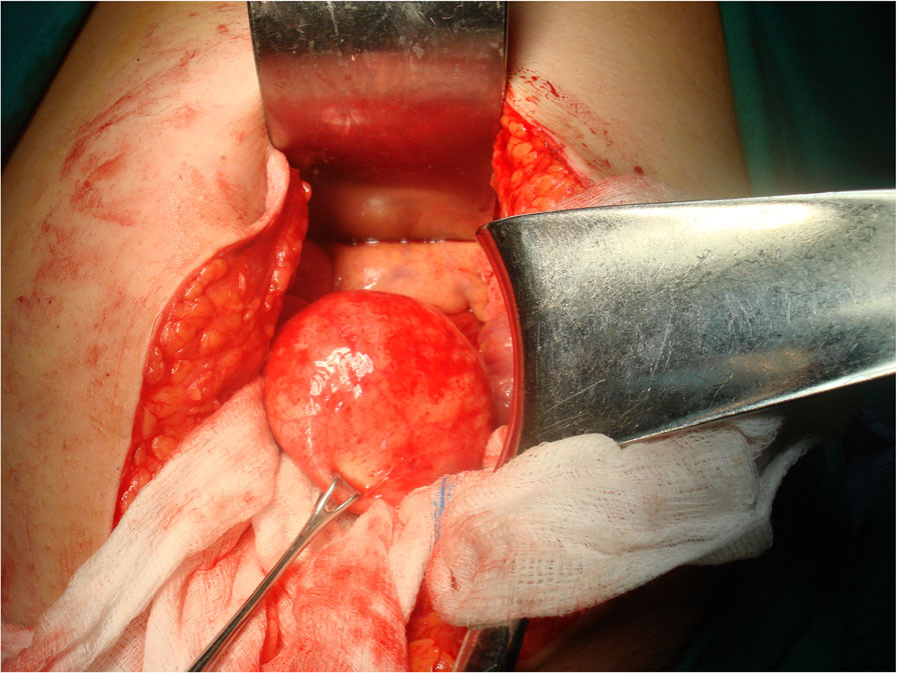
Intraoperative image of mesentery cyst with well-defined wall

**Fig. 4:**
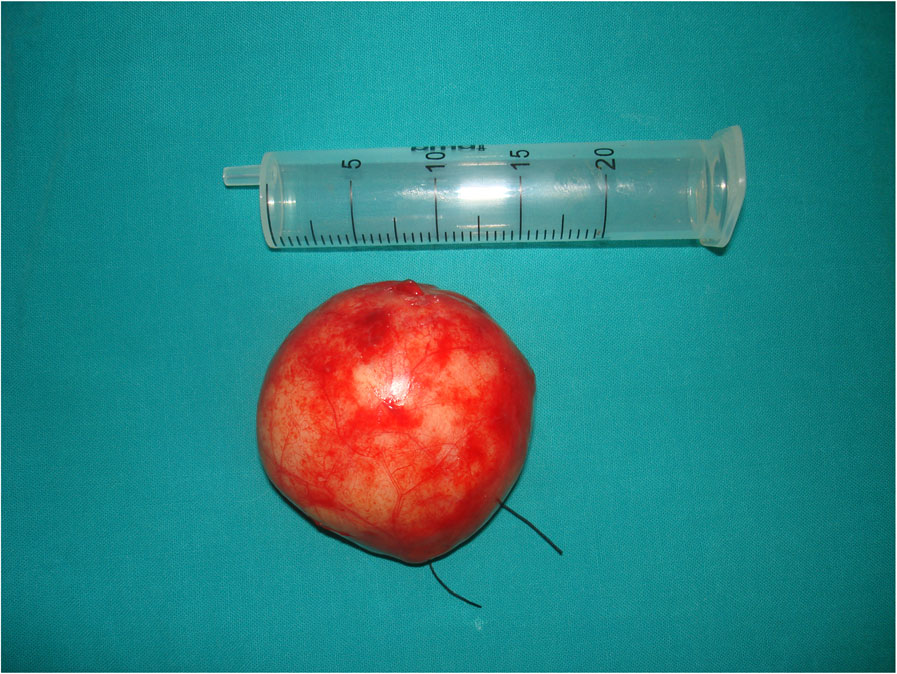
Final specimen

**Fig. 5:**
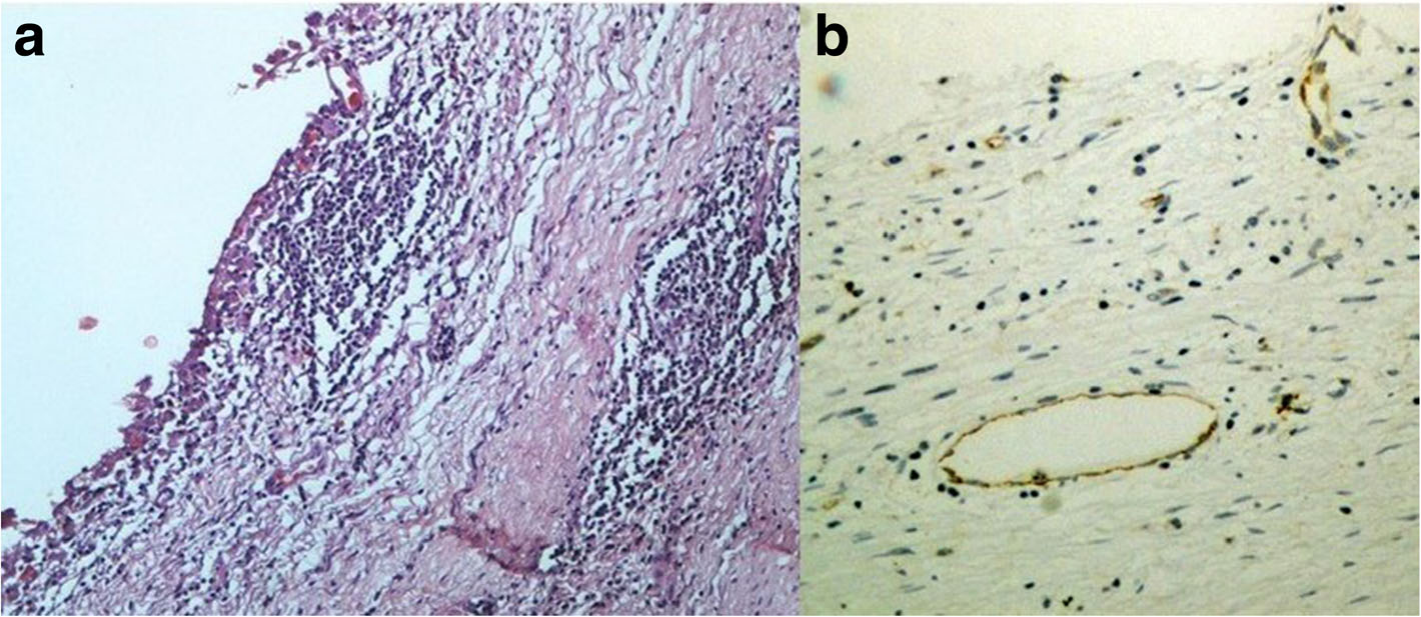
Cyst wall with lymphoid aggregates, hematoxylin and eosin × 100 (**a**) and CD31+ vessels, immunohistochemical × 200 (**b**)

**Fig. 6:**
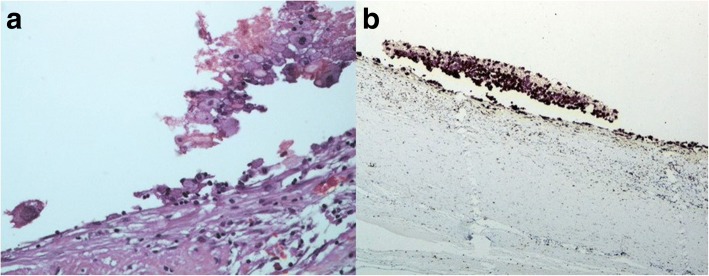
Foreign-body giant cell in the lumen, hematoxylin and eosin × 200 (**a**) as well as CD68+, foamy macrophages, immunohistochemical × 40 (**b**)

We report a case of 38-year-old Greek woman who presented to our Emergency Department complaining of abdominal pain during the last week without any other symptoms. Her clinical history was clear and she had not noticed the occurrence of the same symptoms before. A clinical examination revealed only focal tenderness in the left part of her abdomen. Laboratory results were within normal limits. During an ultrasound examination of her abdomen, an anechoic lesion in her upper left abdomen was revealed. In a further investigation with computed tomography (CT), a well-defined hypodense cystic 7.08 × 6.05 cm mass with mild enhancement was noted (Fig. 2). Surgical approach was decided after a thorough examination and our patient gave her consent for surgery. A cystic lesion sized 7.08 × 6.05 cm appeared between the layers of small bowel mesentery (Fig. 3). The cystic lesion was excised within healthy borders and sent for further pathologic examination (Fig. 4). On macroscopic examination, the cyst sized 7.08 × 6.05 cm was unilocular and contained a white, milk-like viscous fluid (chylous), which was drained out by incision. Histopathological investigation showed a thick fibrous wall, pervaded by chronic inflammatory cells (lymphocytes and plasma cells) and lymphoid aggregates. Variously sized vessels could also be observed while immunohistochemically CD31 (platelet endothelial cell adhesion molecule) was positive (Fig. 5a, b). CD31 is used primarily to demonstrate the presence of endothelial cells and can help to evaluate the degree of tumor angiogenesis. A definitive inner epithelial lining was not found. On the inner surface, multiple aggregates of foamy macrophages as well as focal foreign-body giant cells were present. (Fig. 6a, b) The thickness of the wall varied trivially with small parts of mature fat tissue toward the outer surface, indicating the mesentery. The findings were found to be most consistent with a simple lymphatic (chylous) cyst of the mesentery in combination with features of a non-pancreatic pseudocyst. Her postoperative course was uneventful and patient feeding was started on the second postoperative day. She was discharged on the fifth postoperative day while she had a low-grade fever. A 6-month follow-up with abdomen ultrasound and 1-year CT imaging showed no signs of recurrence.

## Discussion

Although chylous cysts are usually asymptomatic, however, they may give rise to symptoms due to compression of adjacent structures, stretching of the mesentery by rapid expansion, infection, or rupture with hemorrhage [7]. There are cases in which chylous cysts mimicked rupture of abdominal aortic aneurysm and pancreatitis [7, 8]. Symptoms include abdominal distention, vague abdominal pain or even acute abdomen, the presence of a palpable mass, intestinal obstruction, and obstructive uropathy. Cases can also be found incidentally during other surgical procedures and rarely they can be multiple [9]. It has also been estimated that malignant transformation may occur in 3% of such cysts [4, 10].

The specific diagnosis of these lesions is difficult prior to surgery as there are no pathognomonic symptoms or imaging findings. Abdominal radiographs are usually non-diagnostic. Ultrasound, which is often used in the initial evaluation of a suspected abdominal mass, may show a well-defined fluid-filled cystic structure adjacent to bowel loops [5–10]. A fluid-fluid level has been reported as a characteristic finding of these cysts resulting from an upper fluid level due to the chyle and a lower fluid level due to the heavier lymph [11]. A CT and/or MRI scan may additionally demonstrate the fluid attenuation of the lesion, its relationship with the adjacent viscera and vessels, as well as the characteristic chyle-lymph fluid level [12]. A typical feature of mesenteric lymphangioma is a multiloculate mass with homogeneous fluid component [10–13]. Laboratory tests of cyst fluid can determine the biochemical composition while the presence of chylomicrons, cholesterol, and triglycerides is diagnostic for chyle [13]. The differential diagnosis of these cysts includes pancreatic pseudocysts, hemangiomas, endometriosis, loculated ascites (usually tuberculous), peritoneal inclusion cysts, cystic mesenteric panniculitis (sclerosing mesenteritis), hydatid cyst, cystic teratoma, and urogenital cysts [10].

In cases of a large chylous cyst, especially in symptomatic cysts, surgical excision is advised to prevent a potential malignant transformation as well as the development of complications. The preferred technique entails open or even laparoscopic enucleation of the mesenteric cyst; that is, the atraumatic separation of the cyst from the surrounding leaves of mesentery [7–13]. Whenever enucleation cannot be performed safely, due to adhesions of the cyst wall to surrounding mesenteric tissue and/or other structures, a resection of adjacent organs may be necessary (bowel, spleen, pancreatic tail). It has been reported that bowel resection is necessary for only one out of three of the treated adults [14]. Partial excision of cyst, drainage, and deroofing have also been described as potential treatment options; however, the last two options in particular have been associated with a higher likelihood of recurrence and thus are best avoided [11–15]. Endoscopic removal was also referred to in the literature but the method has many limitations [15].

In our case, after initial abdomen exploration, a mass sized 7.08 × 6.05 cm arising from small bowel mesentery was revealed. Our target was the complete excision of the cyst within healthy borders. In case of diverticulitis, open laparotomy approach is the treatment of choice. In this case, laparoscopy was not considered because enucleation of the cyst could not be performed safely due to adhesions of the cyst wall to surrounding mesenteric tissue. The total operative time was 105 minutes and our patient’s postoperative period was uneventful. She was discharged on the fifth postoperative day.

Histopathological examination of the surgical specimen may reveal a unilocular or multilocular cyst, containing a viscous fluid with chylomicrons, cholesterol crystals, and triglycerides (chyle), surrounded by a single layer of flattened mesothelial immunoreactivity cells with cytokeratins and lining a fibrous wall with lymphocytes [4, 10, 12].

The findings of our systematic review including location, gender, size, and publication date are shown in Table 1. The mean age of chylous mesenteric cyst presentation was 50.82 years (range, 22–80) and the male to female ratio was 1.7:1.

**Table 1:** Review of the literature with cases of chylous mesenteric cyst

Author name and reference number	Date	Location	Age	Gender	Clinical presentation	Size (cm)	Current treatment
Pantanowitz and Botero [10]	2000	Small bowel mesentery	39	M	Abdominal discomfort	23 × 15 × 3	Open cystectomy
Ho *et al*. [7]	2002	Small bowel mesentery, aorta	76	M	Abdominal pain, hypotension	5 × 5	Open cystectomy and AAA repair
	2006	Jejunal mesentery	67	M	Abdominal fullness	Unknown	Endoscopically (patient refused surgery)
Miljković *et al*. [13]	2007	Jejunal mesentery	37	M	Abdominal pain, nausea, fever	11 × 14	Open cystectomy
Yasoshima *et al*. [14]	2000	Jejunal mesentery near pancreas	66	M	Asymptomatic	45 × 40	Open cystectomy
Covarelli *et al*. [16]	2008	Jejunal mesentery	62	F	Asymptomatic	Unknown	Open cystectomy
Akwei *et al*. [8]	2009	Mesentery near pancreas head	62	M	Abdominal pain, fever, vomiting, nausea	25 × 15 × 10	Open cystectomy
Tebala *et al*. [1]	2010	Mesentery near cecum	58	M	Chronic abdominal pain, cystic lesion	14 × 12	Laparoscopic cystectomy
Javed *et al*. [12]	2011	Jejunal mesentery near pancreas tail	59	M	Abdominal pain	10 × 10	Open cystectomy
Javed *et al*. [12]	2011	Jejunal mesentery (3 cysts)	35	M	Abdominal distension	Unknown	Jejunectomy
Singh *et al*. [17]	2013	Mesentery near Pancreas tail	22	M	Abdominal pain, Vomiting	Unknown	Open cystectomy
Dioscoridi *et al*. [2]	2014	Jejunal mesentery, Aorta, IMA	30	M	Diffuse abdominal pain	95 × 60	Laparoscopic cystectomy
Wang *et al*. [9]	2014	Small bowel mesentery	80	F	Heavy sweats and shivering	2 × 1.5 × 1	Colectomy for tumor
Lee *et al*. [4]	2016	Jejunal mesentery	34	F	Abdominal pain	10 × 10	Laparoscopic cystectomy
Yoshimitsu *et al*. [18]	2016	Mesentery near pancreas tail	49	F	Asymptomatic	4.9 × 4.2	Laparoscopic cystectomy
This case report	2017	Small bowel mesentery	38	F	Abdominal pain	7.08 × 6.05	Open cystectomy

*AAA* abdominal aortic aneurysm, *F* female, *IMA* inferior mesenteric artery, *M* male

## Conclusions

Chylous cysts represent a diagnostic challenge and they should be considered when a physician encounters an intraabdominal mass. Physical examination and imaging do not always provide a diagnosis and surgical management should be advised due to the potential complications that may develop.
